# Elements of Practical Medicine

**Published:** 1889-06

**Authors:** 


### Reviews of Books.

*Elements of Practical Medicine.* By ALFRED H. CARTER, M.D. Lond. Fifth Edition. London: H. K. LEWIS. 1888.

*The Student's Textbook of the Practice of Medicine.* By ANGEL MONEY, M.D. Lond. London: H. K. LEWIS. 1889.

These two books are intended to fulfil much the same purpose. Dr. Carter's, with a somewhat more humble title than Dr. Money's, is rather more bulky; but owing to difference in type, they contain about an equal amount of matter. And the matter in both cases is excellent.

With so much condensation of a large subject there must be failure here and there. The weak part in Dr. Carter's book is the treatment; but in this, perhaps, there is scarcely any harm to be apprehended, as the author only intends to give "hints as to principles." He makes up for his deficiencies in this respect by providing thirty pages of prescriptions which are to be used for various diseases and symptoms. Notwithstanding the warning he gives against slavishly following these, we cannot help thinking that such a list encourages that objectionable practice of treating the disease rather than the patient.

When, as in Dr. Angel Money's book, the "Practice of Medicine" is given in 434 foolscap octavo pages of fairly open long primer type, it may be thought that the power of condensation can no further go.

But we are bound to say that Dr. Money has done his work well. His descriptions are extremely lucid, and we may safely predict the fulfilment of his hope that the book will be useful, not only to students as an introduction to larger treatises, but also to practitioners in their everyday work. It is a pity that a little more care was not given to the proof-reading, although we should have lost the amusing list of *errata* by which the book is introduced to our notice.

Both these books would have been more acceptable if they had been unaccompanied by the very exact information of the authors' private addresses. Dr. Money also offends by supplying a list of his other publications, including his contributions to medical periodicals.

